# Risky business: A mixed methods study of decision-making regarding COVID-19 risk at a public university in the United States

**DOI:** 10.3389/fpsyg.2022.926664

**Published:** 2022-07-29

**Authors:** Shelley N. Facente, Mariah De Zuzuarregui, Darren Frank, Sarah Gomez-Aladino, Ariel Muñoz, Sabrina Williamson, Emily Wang, Lauren Hunter, Laura Packel, Arthur Reingold, Maya Petersen

**Affiliations:** ^1^Division of Epidemiology and Biostatistics, University of California, Berkeley, Berkeley, CA, United States; ^2^Facente Consulting, Richmond, CA, United States

**Keywords:** COVID-19, risk, qualitative, risk behaviors, students

## Abstract

**Introduction:**

Until vaccines became available in late 2020, our ability to prevent the spread of COVID-19 within countries depended largely on voluntary adherence to mitigation measures. However, individual decision-making regarding acceptable COVID-19 risk is complex. To better understand decision-making regarding COVID-19 risk, we conducted a qualitative substudy within a larger Berkeley COVID-19 Safe Campus Initiative (BCSCI) during the summer of 2020, and completed a mixed-methods analysis of factors influencing decision-making.

**Materials and methods:**

We interviewed 20 participants who tested positive for SARS-CoV-2 and 10 who remained negative, and analyzed quantitative survey data from 3,324 BCSCI participants. The BCSCI study enrolled university-affiliated people living in the local area during summer of 2020, collected data on behaviors and attitudes toward COVID-19, and conducted SARS-CoV-2 testing at baseline and endline.

**Results:**

At baseline, 1362 students (57.5%) and 285 non-students (35.1%) said it had been somewhat or very difficult to comply with COVID-19-related mandates. Most-cited reasons were the need to go out for food/essentials, difficulty of being away from family/friends, and loneliness. Eight interviewees explicitly noted they made decisions partially because of others who may be at high risk. We did not find significant differences between the behaviors of students and non-students.

**Discussion:**

Despite prevailing attitudes about irresponsibility of college students during the COVID-19 pandemic, students in our study demonstrated a commitment to making rational choices about risk behavior, not unlike non-students around them. Decision-making was driven by perceived susceptibility to severe disease, need for social interaction, and concern about risk to others. A harm reduction public health approach may be beneficial.

## Introduction

Until vaccines became available in late 2020, our ability to prevent the spread of coronavirus disease 2019 (COVID-19) within countries depended largely on voluntary adherence to mitigation measures (Clark et al., 2020; Fischhoff, 2020). However, individual decision-making regarding acceptable risk of COVID-19 is complex; simply educating the general public about mitigation strategies and issuing government recommendations or regulations may be insufficient to achieve broad scale behavior change on the individual level.

Three main themes around decision-making have become evident over the course of the COVID-19 pandemic. First, so-called government-initiated “lockdowns” or “shelter-in-place” orders have raised concerns about the mental health implications of extended isolation, and made clear the importance of balancing individuals’ need for social connection with their desire to stay uninfected by SARS-CoV-2 (Brooks et al., 2020; Codagnone et al., 2020; Burrai et al., 2021; Kornilaki, 2021; Rolón et al., 2021; Romero-Rivas and Rodriguez-Cuadrado, 2021; Saban et al., 2021). One theoretical framework that is helpful for understanding individual decision-making around health-related risk-taking is the Health Belief Model (Badr et al., 2021; Rabin and Dutra, 2021), which posits that individuals choose behaviors based on a balance of their perceived susceptibility to an illness and the perceived benefits and barriers to taking action to prevent that illness. Prior research has explored the role of perceived susceptibility to COVID-19 among people in the United States (US) (Niemi et al., 2021); whether individuals have had personal experience with COVID-19 among friends or family has an impact on their perception of disease severity, along with personal susceptibility (Cherry et al., 2021). Perceived benefits and barriers of complying fully with COVID-19 community mitigation strategies, such as staying home except for essential activities, wearing a face covering in public, and socially distancing from others have also been documented (Mallinas et al., 2021), including the mixed impact of moral condemnation of those who are not compliant with regulations or are seen as taking excess risks (Henderson and Schnall, 2021).

A second theme relates to the role of individualism vs. collectivism in decision-making during COVID-19, with prior research indicating that some are motivated by protecting others (i.e., a sense of community responsibility), while others are primarily motivated by self-protection, in which case perceived personal susceptibility is a much more influential factor (Comfort et al., 2020; Burrai et al., 2021; Kumano et al., 2021; Lu et al., 2021; West et al., 2021). Third, there is clear evidence that health behaviors related to COVID-19 have become increasingly politicized, especially in the US (Byrd and Białek, 2021; Rabin and Dutra, 2021; Tan et al., 2021; Testa et al., 2021), with the implication that perceived benefits and barriers to taking action are driven largely by political affiliation and politically driven messaging. Political affiliation or related affinity toward others with similar beliefs can also impact individuals’ beliefs toward science as a driving factor in decision-making and social norms around compliance with government regulations or other rules, both of which have been demonstrated to impact COVID-19-related risk behavior (Bicchieri et al., 2021; Gelfand et al., 2021).

To better understand these dynamics and their influence over decision-making regarding COVID-19 risk behaviors, we conducted a qualitative substudy nested within the larger Berkeley COVID-19 Safe Campus Initiative (BCSCI) study during the summer of 2020.

## Materials and methods

The original BCSCI included 3,324 students, faculty, staff, and essential workers affiliated with the University of California, Berkeley campus who enrolled in June 2020 by providing written consent, then completing a baseline survey and providing specimens for polymerase chain reaction (PCR) and antibody testing for SARS-CoV-2. Participants were then asked to complete weekly surveys related to COVID-19 exposure and risk behaviors, in addition to a longer endline survey at study close in August 2020, when most participants were also asked to provide another set of specimens for PCR and antibody SARS-CoV-2 testing. More details about the methods of the main BCSCI study have been previously reported (Packel et al., 2021).

After the endline survey was closed, study staff recruited 20 participants who had tested SARS-CoV-2 positive and 10 who had tested SARS-CoV-2 negative through the BCSCI study to participate in a 1-hour in-depth interview as part of a qualitative substudy. A random sample of 10 people having tested positive for SARS-CoV-2 at least once was generated from the BCSCI participant list, and those participants were sent an email inviting them to participate in the qualitative substudy. Up to two follow-up phone calls or text messages were sent before that participant was considered non-responsive, at which time a new random sample of 10 people was generated and this sequence was repeated until a total of 20 substudy participants was reached. Once 20 SARS-CoV-2 positive participants were enrolled in the substudy, 10 participants with consistently negative SARS-CoV-2 results were randomly sampled from the main study, matched 2:1 with those testing positive on campus role (i.e., student, essential worker, faculty, or staff), sex, age, race/ethnicity, time of main study enrollment, and residence in group housing. These negative participants were recruited in a similar fashion until a total of 10 consistently SARS-CoV-2 negative participants were enrolled.

People who indicated interest in participating in the qualitative substudy were emailed a link to an electronic informed consent form and brief survey *via* Research Electronic Data Capture (REDCap) (Harris et al., 2009, 2019), and then participated in Zoom-based video interviews using a semi-structured interview guide (see Supplementary Material). Transcripts were automatically generated through the Zoom platform; on three occasions, failure to automatically transcribe led to the audio file being transcribed manually or *via* Rev.com. Transcripts were iteratively coded by study team members (SF, MD, DF, SW, AM, and SG-A) in Dedoose Version 8.3.45 [SocioCultural Research Consultants, LLC, Los Angeles, CA, United States]. Each transcript was double-coded, with discrepancies resolved by team discussion and consensus. Fully coded transcripts were analyzed using immersion and crystallization techniques (Borkan, 1999).

This research was approved by the UC Berkeley Committee for the Protection of Human Subjects (CPHS), protocols #2020-07-13461, #2020-06-13349, #2020-05-13261, and #2020-04-13238.

## Results

Themes from the qualitative interviews and related questions on the baseline or endline surveys generally fell into five categories: perspectives on mitigation mandates, compliance with those mandates, perspectives on others’ compliance/risk-taking, factors affecting risk-related decision-making, and what participants wished they had done differently with regard to risk behavior, upon further reflection.

### Perspectives on mitigation mandates

During the qualitative interviews, four respondents specifically noted that—compared to other regions they had seen or heard about—most people in the local area seemed to be visibly masking and distancing while in public, especially when in stores or other area businesses. In the baseline survey, 2014 students (84.5%) thought that COVID-19 community mitigation measures asked of them by the local health department were “very important” and 348 (14.6%) thought they were somewhat important, compared to 771 (96.4%) of faculty, staff, and essential workers (referred to from here forward as “non-students”) who thought the mitigation measures were “very important” and 38 (4.8%) thought they were “somewhat important.” In the qualitative interviews, two people (both students who tested COVID-19 positive) specifically noted they thought the Bay Area regulations were “too much.” Eight people (four positive, four negative; six students and two non-students) said they thought the regulations were “not enough.” Sixteen participants (10 positive, six negative; 14 students and two non-students) said they thought the regulations were “just right.”

### Compliance with mitigation mandates

In the baseline survey, 1,006 students (42.5%) and 528 non-students (64.9%) said it was not at all difficult for them to comply with COVID-19 mandates, while 1,237 students (52.2%) and 268 non-students (33.0%) said it had been somewhat difficult for them to comply, and 125 students (5.3%) and 17 non-students (2.1%) said it had been very difficult for them to comply. For those who said it had been somewhat or very difficult to comply, the most-often cited reason was the need to go out for food or other essentials, with the second and third-most common reasons being difficulty of being away from family/friends and loneliness (see Table 1). This finding was in alignment with responses to a baseline survey question about why people had left the house in the past 7 days (3 months into local shelter-in-place orders), where 1,477 people (44.4%) said they had left to socialize with people outside their household. When asked on the endline survey whether it is unreasonable to expect that students will not socialize during the pandemic, 1,335 students (68.1%) strongly agreed or agreed with that statement and only 318 (16.2%) disagreed or strongly disagreed.

**TABLE 1:** Stated reasons for difficulty complying with COVID-19 mandates, among participants who said it had been very (*n* = 142) or somewhat (*n* = 1505) difficult to comply.

It’s difficult for me to comply because…	It has been very difficult for me to comply	It has been somewhat difficult for me to comply
I can’t work from home	37 (29.6)%	261 (21.1)%
I don’t believe measures are effective	7 (4.9)%	48 (3.2)%
I need childcare	12 (8.5)%	60 (4)%
It’s too hard for me to be away from family/friends	96 (67.6)%	765 (50.8)%
I’m lonely	93 (65.5)%	638 (42.4)%
I need to go out for food or other essentials	107 (75.4)%	1175 (78.1)%
Another reason	26 (18.3)%	191 (12.7)%

While “shelter-in-place” orders were aimed at reducing risk through limiting in-person interactions, in-person interactions are not inherently high risk for transmission of SARS-CoV-2 if other mitigation strategies are utilized, such as masking. While at baseline 2,916 people (98.7%) always wore a mask while shopping, only 966 (37.8%) said they always wore a mask while socializing with people outside their household, and 251 (9.8%) said they never wore a mask while socializing (see Table 2). However, at endline 1,102 students (56.2%) and 430 non-students (61.8%) said they strongly agreed or agreed that it was reasonable to expect students to wear a mask while socializing, and only 602 students (30.7%) and 196 non-students (28.2%) disagreed or strongly disagreed with this.

**TABLE 2:** Frequency of wearing a mask or staying at least six feet away from all other people (“social distancing”) while in public.

Mitigation strategy	Always	Sometimes	Never
Wore a mask while at work outside the home	1018 (72.7)%	233 (16.6)%	150 (10.7)%
Wore a mask while shopping	2916 (98.7)%	32 (1.1)%	7 (0.2)%
Wore a mask while outside for exercise/leisure	766 (25.6)%	1747 (58.4)%	478 (16)%
Wore a mask while socializing	966 (37.8)%	1341 (52.4)%	251 (9.8)%
Social distancing while in public	1957 (61.5)%	1218 (38.3)%	9 (0.3)%

In the qualitative interviews, five people—including four who tested positive for SARS-CoV-2—said they thought they had been closely adhering to all public health regulations. One student explained, “*I was pretty surprised that I tested positive, because I thought that I was following pretty much all of the public health guidelines as well as I could*…*I was essentially part of [my in-laws at high risk for severe disease’s] quarantine bubble, which meant being very careful, or at least trying to be very careful not to pick up the virus anywhere.”* Another student highlighted that their decision-making was based on what was allowed per the changing health orders: *“I’m still not doing anything that I would consider, like, really non-essential. I mean, golf is like hiking. It’s open. I didn’t golf when golf wasn’t allowed. You know, when they said, like, “Only go outside for essential activities,” I am not going outside for personal activities.”*

Eight people—including four who tested positive (all different from those referred to above)—said that as of the time of the interview (August/Sept 2020) they were *always* wearing a mask outside their home, even when outside, unless they were sure no one was around (i.e., on a hike in the woods without anyone in sight). One essential worker explained:

*“The only time I’m without the mask*…*like, I’ll run to the car really quick, if it was like half a block away and there’s nobody in sight. And I just, for whatever reason, would want some fresh air on my face. But otherwise, you know, we’re always masked at work, wear masks. When we go pick up our kids from daycare, masks at the grocery store, we’re always masked. And in terms of social distancing, we don’t really go near anybody else, just within our own household.”*

A student raised the concept of collective responsibility, stating: *“I feel like it’s almost like my job, even though I don’t feel like I could get it, making sure people around me feel comfortable, so that when I go on a walk, even though I’m not worried about giving or getting it, I still will put on a mask. When I go by someone because I kind of feel like that’s just part of the social contract.”*

However, three people—all of whom tested SARS-CoV-2 positive—described making calculated exceptions to rules about masking or social distancing, as one student’s comments exemplified: *“[In places that are] public, like a grocery store or park, I always wear a mask and always socially distance. However, my close friends*…*in college, if they’re okay with*…*they will ask each other for consent, like whether they’re OK with hanging out and it’s just a small group, but we all know that none of us are at risk, none of us are immunocompromised, and so we do study together, and we do hang out, but all of us take it seriously.”*

### Perspective on others’ risk-taking

Most of the people in the qualitative substudy had a positive perspective on people’s adherence to Bay Area COVID-19 mandates (i.e., thought most people were masking and generally following regulations). However, three people (two SARS-CoV-2 positive and one negative) said they didn’t think others could do what was needed to stay safe, as one student explained, “*I would say it’s shifted my faith in humanity as a whole, and particularly [my faith] in America, of being like, why can’t we just be more responsible, you know?”* Two people who had tested SARS-CoV-2 positive expressed anger or resentment about the fact that despite working hard to adhere to regulations they were infected, when so many others they saw were doing far riskier things. One student alluded to their own experience of “COVID fatigue”:

*“I would say I was definitely like among the*…*best adherence, and then, you know, around the middle of the summer, just got a bit more lax when things were getting—you know, there was a certain point where I felt saturated with all the information about how bad everything was. And so I just wasn’t really consuming much news about the pandemic around the time that I got infected, and so I think that caused me to become less compliant with the regulations. Like for example, I did do some outdoor playing out with some friends, where we were not wearing masks. I mean, we were like, you know, 6 or 8 feet apart sitting in the park. But I think the rule was you’re supposed to wear masks. And then there was one more time around that weekend as well, where I was inside without masks, which I figure is probably where I got it.”*

Three other participants (two SARS-CoV-2 negative and one positive) expressed a sense of anxiety watching others take risks, as one student elaborated: *“It’s really stressful to hear that frats are having parties near campus when I know that I’m working so hard and not seeing my friends. And it feels like other people aren’t taking this with the same level of seriousness*…*I want to be able to go back to seeing my friends! And I wish I could graduate in person. I want everyone to be as careful as I’m being [so this can happen], and it’s stressful seeing people not doing that.”* Another student noted, *“I see people, like, on TV, being in a gathering and it makes me anxious. Don’t do that! So I feel like behaviorally I’m probably being conditioned to be more nervous about walking by people or being near people, so I can see that having a lasting impact, even if I’m like, “OK, rationally, this is fine.””*

### Decision-making regarding COVID-19 risk

Without fail, participants in the qualitative interviews described very deliberate decision-making about the risks they were willing to take during the early months of the pandemic. Eight people (five SARS-CoV-2 positive, three negative) said they consistently wore masks around strangers—such as in stores—but not outdoors when they were far away from others, or when they were around people they knew well; two (one positive, one negative) specifically noted they would cross the street to avoid strangers when out and about. Three people (one positive, two negative) said they would only go to stores for food/essentials, and would strategize the place or time they would go to reduce contact with others.

When it came to spending time with others they knew well, five people (four of whom tested positive) said they had decided to continue seeing friends or family outside their household because they had conversations with them about risks and behaviors and, as one said, “know they’re pretty safe.” Another four people (one positive, three negative) said they were willing to see friends while the shelter-in-place order was in effect, but would always take steps to mitigate risk, such as only being outside, distanced, and masked; not sharing food/plates/utensils; or being indoors but with open windows and distancing.

Eight people in the qualitative interviews (four negative, four positive) explicitly noted that they made decisions not just regarding risk to themselves, but also risk to others around them. One student explained, “*It sucks [to have conversations with people about the prior risks they’ve taken] but I think it’s a reasonable thing to do right now. To watch out for each other, because at the end of the day, especially if you’re living with people, it’s not just you that you’re responsible for, it’s everyone else you’re living with. And I would just feel terrible if I gave COVID to everyone in this house!”* Another student noted, *“We avoid doing things [like staying home and instead using a food or grocery delivery service] if that just means someone else will need to do them for us, because that would just put someone else at risk. We’re aware that it’s usually people who don’t have a choice but to work in vulnerable situations. So we try to avoid situations like that.”*

Importantly, two students noted that they felt trapped in situations that were much higher-risk than they would otherwise choose, such as having no option other than taking public transportation or living with other students. One described,

“*I’m broke as hell*…*and so*…*I basically had no other option than to live in [Greek housing] because it was so cheap*…*I felt like I basically had no control over the actions of other people because I was, you know*…*the house is not my personal house. And there are too many people to try and corral. So I ended up sort of like I was, like, “Well, this is just something that I have to deal with because, you know, rent is so cheap and I have basically nowhere else to go.””*

### What people wish they had done differently

Five people (four of whom tested SARS-CoV-2 positive), identified a specific thing they had done to put themselves at risk that, had they been able to do it over again, they wouldn’t have done. Another (who remained negative as of September 2020) said they wished they had left group housing on campus and gone home to live with family outside of the local area, which would have been safer and less stressful. Three people (two of whom tested positive) described being more cavalier early on, when they underestimated the severity of COVID-19, as one described: “*I think maybe toward the beginning I didn’t*…*I would see a few friends in the beginning of everything*…*not really realizing how easily I could have gotten it, and how quickly. And how detrimental that would have been, if I gave it to my family. I wasn’t really thinking of that, because no one I had known ever had gotten it.”*

Five people who tested positive said they would have changed nothing, despite having contracted SARS-CoV-2—they thought they did everything as well as could be expected, and unfortunately this is a virus that is very easily transmitted (i.e., they were either unlucky or it was inevitable). One student who tested positive specifically said they wouldn’t have trusted others so much, and would have directly asked more specific questions about people’s behaviors: “*[I would have stopped] assuming that everybody else is conforming with the recommended suggestions to the same degree that I am.”*

Notably, when asked if they would have done anything differently during the first 6 months of the pandemic, four people who tested negative and one who tested positive said they wished they’d “gotten out more,” and/or found ways to see more people despite the shelter-in-place order, given the toll it took on their mental health to be isolated for so long.

Table 3 provides an overview of the main themes from this analysis, stratified by SARS-CoV-2 test result history at the close of the study.

**TABLE 3:** Comments or reflections proactively raised by participants during interviews (i.e., these themes were not responses to specific survey-style questions, but rather were volunteered by participants).

Comment or reflection	Number out of those who tested positive (*n* = 20)	Number out of those who consistently tested negative (*n* = 10)
Believed that Bay Area regulations to prevent transmission of COVID-19 were “too much”	2	0
Believed that Bay Area regulations to prevent transmission of COVID-19 were “just right”	10	6
Believed that Bay Area regulations to prevent transmission of COVID-19 were “not enough”	4	4
Believed that they had been closely adhering to all public health regulations	4	1
Reported *always* wearing a mask outside their home in August/Sept 2020, even when outside, unless they were sure no one was around	4	4
Reported consistently wearing masks around strangers, but not outdoors or when around people they knew well	5	3
Reported making calculated exceptions to official rules or recommendations about masking or social distancing	3	0
Reported continuing to see friends or family outside their household during early shelter-in-place orders without masking or other mitigation strategies because of discussions implying a shared commitment to protective behavior (i.e., “they’re pretty safe”)	4	1
Reported continuing to see friends or family outside their household during early shelter-in-place orders but only with mitigation strategies in place such as masking, outdoor interactions, and/or open windows and social distancing	1	3
Reporting making decisions about COVID-19-related behaviors to influence not just personal risk but also risk to others around them	4	4
Identified a specific incident of high risk that they would not have done if able to do it over again	4	1
Believed others around them were unable or unwilling to do what was needed to keep themselves and others safe during the pandemic	2	1
Described a sense of anxiety watching others taking COVID-19-related risks	1	2
Reported wishing they had “gotten out more” and/or seen more people during the first 6 months of the pandemic, given the mental health toll of isolation	1	4

## Discussion

Much ado has been made about the behaviors of college students during the COVID-19 pandemic—particularly about self-indulgent, irresponsible behavior that was likely to spread COVID-19 among themselves and put others in the community at risk. However, students in our study demonstrated quite a different reality: they were making rational choices about risk behavior, not unlike non-students around them. Questions about risk behaviors in the BSCSI surveys did not, on the whole, show significant differences between the behaviors of students and non-students. Choices about risk-taking in the early months of the pandemic—at a time when community transmission in the local area was spiking—were driven by factors outlined by the Health Belief Model: participants discussed their assessment of their own risks of severe disease (perceived susceptibility), their need for social interaction during their college experience (perceived barriers to self-isolation), and their concern about being a risk to others (perceived benefits of complying with strategies). Even those who tested positive for SARS-CoV-2 made strategic decisions regarding risk based on the available information and their personal weighing of risks and benefits. These same decision-making strategies may also affect students’ decisions whether to be vaccinated against COVID-19 as an additional prevention option.

A focus on hospitalization and death as a marker of the main negative effects of SARS-CoV-2 infection may have led to a low perceived susceptibility among young people, and therefore a greater willingness to take risks. What has become known as “long COVID” has, in fact, been shown to be a considerable risk for those in this age group with COVID-19 (Walsh-Messinger et al., 2020; Hageman, 2021), although it has not been a prominent part of public health messaging around this disease, and was not mentioned by a single interview participant. Especially given the generally low perception of risk that most students in this substudy noted, public health messaging relying on shaming students into compliance is not likely to be effective. While there is limited research on COVID-19 messaging strategies with students, other studies related to obesity (Simpson et al., 2019), sexual health (Brickman and Willoughby, 2017), tobacco (Rath et al., 2019), and drug use (Watson et al., 2019; Ventresca et al., 2021) have found that young adults often reject moralizing, negative public health messaging. On the other hand, messaging that attempts to influence social norms (Agranov et al., 2021) or amplify the clear recommendations of experts who have no likely conflicts of interest (Bogliacino et al., 2021) may be much more productive.

Ultimately, study participants on this university campus expressed a need to balance a desire to stay safe from COVID-19 with a need to counteract loneliness through physical and emotional socialization—particularly for extroverts (Landmann and Rohmann, 2021; Liu et al., 2021). It was notable that four people in our study who tested SARS-CoV-2 positive didn’t have any regrets, emphasizing that they would change nothing about their risk-related decisions if they could do it again.

This qualitative study had a number of limitations. First, as with all qualitative studies, the small number of participants limits the generalizability of findings, and the unique nature of this study population (an academic population in the San Francisco Bay Area of California) may further limit generalizability to other settings. However, the themes that arose from this substudy were largely in concordance with other studies about risk-taking related to COVID-19 or other infectious diseases, and are a useful starting place for further exploration of these dynamics with a larger sample size in future research. Second, there was likely selection bias of interview participants—while those sent invitations to participate in the study were chosen at random, acceptances were not; it is likely that those who agreed to participate in interviews were more intentional—and potentially more cautious—about their COVID-19-related risk-taking than those who declined. Third, those who participated may have censored their discussion of risk behaviors or sense of responsibility toward others in the community, resulting in social desirability bias in findings. Fourth, this study took place early in the pandemic, and findings may not apply to people’s motivations or decision-making at later phases of the pandemic. Future investigation would be useful regarding the factors that influence decision-making in later stages of the pandemic—especially of those related to personal experience with self or family members contracting COVID-19 and the acute or long-term impacts of this. Further research is also warranted into the specifics of living situations and the impact on risk decision-making, e.g., the differing choices of those living alone, living with non-familial roommates, living with extended family who may be at greater risk of severe COVID-19 disease, etc., as these factors could not be thoroughly explored with our small and limited qualitative sample.

We know from other diseases or health risks that personal behavioral decision-making is very complex—not simply about doing what’s good and avoiding what’s bad. This is also true for COVID-19, requiring public health practitioners to offer clear messages that address the complexities of making these decisions, especially for young people. A harm reduction (rather than abstinence-based) approach to public health strategies may be beneficial (Packel et al., 2021), although this has not been a typical framework during the COVID-19 pandemic (Marcus, 2020; Gravett and Marrazzo, 2021). One option would be less focus on firm rules of green/yellow/red behaviors, and more focus on conversation and risk negotiation with others. Students in this study repeatedly raised the importance of frank and honest discussions that would allow them to socialize more safely with others, rather than depriving oneself completely of social contact. Starting with the premise that people on a university campus are rational beings who want to protect themselves and others—assuming the tradeoffs are not so great as to tip the balance toward non-compliance with mandates—will allow public health messaging during this and future pandemics to resonate more strongly, and possibly be more effective.

## Data availability statement

De-identified transcripts supporting the conclusions of this article will be made available by the authors, without undue reservation.

## Ethics statement

The studies involving human participants were reviewed and approved by UC Berkeley Committee for the Protection of Human Subjects (CPHS), protocols #2020-07-13461, #2020-06-13349, #2020-05-13261, and #2020-04-13238. The participants provided their written informed consent to participate in this study.

## Author contributions

SF conducted the qualitative and quantitative analysis and wrote the first draft of the manuscript. MD, DF, SG-A, AM, and EW conducted qualitative interviews. MD, DF, SG-A, SW, and SF coded qualitative interviews for analysis. SF, LH, LP, AR, and MP provided supervision and oversight of the larger BCSCI study. All authors reviewed and revised the draft manuscript and approved the submitted version.
