# Supplementary material for: Risky business: A mixed methods study of decision-making regarding COVID-19 risk at a public university in the United States
Source: Front Psychol. 2022 Jul 29;13:926664. doi: 10.3389/fpsyg.2022.926664 (PMC9372553; doi:10.3389/fpsyg.2022.926664)
Supplement: Supplementary file 1 [file Data_Sheet_1.PDF]

SUPPLEMENTAL MATERIAL

SEMI-STRUCTURED INTERVIEW GUIDE – PEOPLE WHO TESTED POSITIVE

INTRO: *Hi, thanks so much for agreeing to do this interview.*

- *My name is [Name] and I am a graduate student researcher working on the studies that are part of the Berkeley COVID-19 Safe Campus Initiative.*
- *I have a handful of questions to ask you today, but this is really meant to be a conversation more than anything so it's OK if your answers aren't exactly in line with the questions – if my questions remind you of an important point, feel free to share.*
- *You can ask for clarification of my questions, and there will be time at the end for you to add anything else that I didn't ask about. You are also welcome to tell me you'd prefer not to answer any of the questions I ask and I'll move on to the next question.*
- *As it said in the consent form you signed, I'm going to video (or audio) record this today, but only study staff will see the video, and if something you say is included in anything we share publicly we will take care to not share any information that could identify you.*
- *As a last point, we didn't say this when we reached out to schedule this because we wanted to be careful to protect your confidentiality, but we've reached out to you for this interview because you tested positive for COVID-19 during the study, so a lot of my questions will have to do with your experiences of testing positive.*
- *Do you have any questions before I start the recording and dive into my first question?*

1. How is your health now? Have you had any medical complications as a result of COVID-19, as far as you know?

2. What are the different ways you've gotten information about risk for COVID-19 during this pandemic?

    - [*Probe: Word of mouth / talking to friends? Social media (FB, IG, Reddit, etc.)? Mainstream media? Official government communications or websites? Official University communications?*]

2. When did you find out you were infected with the virus that causes COVID-19? How did you find out?

    - [*Probe: What worked well, or not well, about the way you were told?*]

3. What led you to decide to get a test for COVID-19 through this study? Were you primarily interested in being in a study, or was the opportunity for testing the reason you joined the study?

- **[*Probe: For each of the times you got a test, were any of them not because the study asked you to?***

    1. ***If so, what prompted you to do that?***

2. ***If no, under what conditions might you decide to go for a test on your own?***

- ***For people who were "randomly" selected for testing through the study: What was your reaction when you got the message asking you to come in for randomly selected testing?*]**

4. **[*Students and Essential Workers only:*] Given your experience so far with the Berkeley COVID-19 Safe Campus study, would you sign up for a system like this (where you had access to testing, filled out a brief survey every day, and would potentially be contacted for extra testing based on information you provided) if there wasn't a monetary incentive for study participation?**

5. **What has your experience been with the testing provided through the study?**

- **[*Probe: Did you know you could schedule a test at any time without giving a reason?***

    1. ***If so, what did you think about that?***

    2. ***If not, were there additional times you would have chosen to get another test?***

- ***How did this system of testing via UHS compare to other types of testing for COVID-19, if you know about any other types of testing?***

- ***Is there anything that worked particularly well, or that you particularly didn't like, about this testing system?*]**

6. **What happened after you found out your result?**

- **[*Probe:* <u>*When were you asked to isolate?*</u>**

- ***What was your experience with isolation like?***

- ***Were you able to use a separate bathroom and/or bedroom while isolating? What support did you receive?***

- **<u>*What additional (material or emotional) support would have been helpful?*</u>**

- ***What was your experience with contact tracing like?***

- **<u>*When were you contacted and asked about whom you might have come into contact with?*</u>**

- ***Were you comfortable discussing your situation and providing info about your contacts?***

- ***Did you have any concerns with contact tracing?***

- ***Was there anything that would have made the contact tracing process easier?*]**

7. Can you describe some of the impacts of testing positive for COVID-19? How did it affect you?

    - [*Probe: Who did you tell? Did anything especially upsetting happen? Did anything surprising happen?*

8. What were you most worried about when you found out you were positive for COVID-19?

    - [*Probe: Did any of those fears come true?*]

9. Overall, how would you say your mental health/well-being has been, related to the COVID-19 pandemic? Even before testing positive, did you find yourself very worried or stressed about anything, and if so, what? What tools (if any) have you found to help you be resilient in the face of it all?

    - [*Probe: What are some of the ways you have coped with your worries?*
    - *How have you made decisions about the level of risk for COVID-19 you were willing to tolerate, as a trade-off for various activities*]

11. Is there anything you would have done differently during this pandemic, now that you know you were infected with COVID-19?

    - [*Probe: How would you rate your level of adherence to regulations around social distancing or facial coverings, compared to others in the Bay Area?*
    - *Do you think these regulations were too much? Not enough? Just right?*]

12. Is there anything I didn't ask that you think it would be important for me to know?

RESOURCES:

Students can call UHS Social Services at 510-642-6074 (leave a voicemail and someone will call you back) or outside of 10-4 M-F they can call the after hours support line at (855) 817-5667 or (877) 211-3686.

Faculty/Staff can call Employee Assistance (formerly CARE Services) at (510) 643-7754, and after hours can leave a confidential message 24-hours a day, which will be returned the next business day. For something more urgent, they can call the 24-hour Community Crisis Line at (800) 309-2131.

SEMI-STRUCTURED INTERVIEW GUIDE – PEOPLE CONSISTENTLY TESTING NEGATIVE

INTRO: *Hi, thanks so much for agreeing to do this interview.*

- *My name is [Name] and I am a graduate student researcher working on the studies that are part of the Berkeley COVID-19 Safe Campus Initiative.*
- *I have a handful of questions to ask you today, but this is really meant to be a conversation more than anything so it's OK if your answers aren't exactly in line with the questions – if my questions remind you of an important point, feel free to share.*
- *You can ask for clarification of my questions, and there will be time at the end for you to add anything else that I didn't ask about. You are also welcome to tell me you'd prefer not to answer any of the questions I ask and I'll move on to the next question.*
- *As it said in the consent form you signed, I'm going to video (or audio) record this today, but only study staff will see the video, and if something you say is included in anything we share publicly we will take care to not share any information that could identify you.*
- *As a last point, we've reached out to you for this interview because you tested negative for COVID-19 during the study, so a lot of my questions will have to do with your experiences of testing negative during this pandemic.*
- *Do you have any questions before I start the recording and dive into my first question?*

1. What are the different ways you've gotten information about risk for COVID-19 during this pandemic? [*Probe: Word of mouth / talking to friends? Social media (FB, IG, Reddit, etc.)? Mainstream media? Official government communications or websites? Official University communications?*]

2. **What led you to decide to get a test for COVID-19 through this study? Were you primarily interested in being in a study, or was the opportunity for testing the reason you joined the study?**

    - **[*Probe: Why (and where) have you accessed testing in the past / outside of the study, if at all?***

    - ***Have you been able to access testing for COVID-19 anytime you wanted it? If not, what were the barriers?*]**

3. Can you describe the testing experiences you've had to date?

    - [*Probe: For each of the times you got a test, were any of them not because the study asked you to?*

        - *If so, what prompted you to do that?*

        - *If no, under what conditions might you decide to go for a test on your own?*

    - *For people who were "randomly" selected for testing through the study: What was your reaction when you got the message asking you to come in for randomly selected testing?*

4. **What has your experience been with the testing provided through the study?**

- **[*Probe: Did you know you could schedule a test at any time without giving a reason?***
  - ***If so, what did you think about that?***
  - ***If not, were there additional times you would have chosen to get another test?***
- ***How did this system of testing via UHS compare to other types of testing for COVID-19, if you know about any other types of testing?***
- ***Is there anything that worked particularly well, or that you particularly didn't like, about this testing system?*]**

5. **[*Students and Essential Workers only:*] Given your experience so far with this study, would you sign up for a system like this (where you had access to testing, filled out a brief survey every day, and would potentially be contacted for extra testing based on information you provided) if there wasn't an incentive for study participation?**

6. **When was the most recent time you tested negative for the virus that causes COVID-19? How did you find out your results?**

- **[*Probe: What worked well, or not well, about the way you were told your results?***
- ***<u>How were the negative test results communicated to you?</u>***
- ***<u>Was it made clear to you that a negative test result did not mean you were definitely uninfected with the virus that causes COVID-19?</u>*]**

7. **Can you describe some of the impacts of testing negative for COVID-19? How did it affect you? [*Probe: Did you make any changes in your behavior as a result of testing negative?*]**

8. What are your biggest worries about the possibility of testing positive for COVID-19?

9. Overall, how would you say your mental health/well-being has been, related to the COVID-19 pandemic? Have you found yourself very worried or stressed about anything, and if so, what? What tools (if any) have you found to help you be resilient in the face of it all?

- [*Probe: What are some of the ways you have coped with your worries?*

- *How have you made decisions about the level of risk for COVID-19 you were willing to tolerate, as a trade-off for various activities*]

10. Have you known anyone close to you who tested positive for COVID-19? Do you know anything about what their experience has been like?

11. Is there anything you wish you had done differently during this pandemic, so far?

    - [*Probe: How would you rate your level of adherence to regulations around social distancing or facial coverings, compared to others in the Bay Area?*
    - *Do you think these regulations were too much? Not enough? Just right?*]

12. What do you think about the various measures that UC Berkeley has put into place to try to protect people against COVID-19 transmission? [*Probe: What do you think of the daily survey to attest that you are symptom-free before going into a building? What have you observed regarding social distancing and facial coverings on campus? How do you feel about any cleaning regimens, etc. that you're aware of?*]

13. Is there anything I didn't ask that you think it would be important for me to know?

RESOURCES:

Students can call UHS Social Services at 510-642-6074 (leave a voicemail and someone will call you back) or outside of 10-4 M-F they can call the after hours support line at (855) 817-5667 or (877) 211-3686.

Faculty/Staff can call Employee Assistance (formerly CARE Services) at (510) 643-7754, and after hours can leave a confidential message 24-hours a day, which will be returned the next business day. For something more urgent, they can call the 24-hour Community Crisis Line at (800) 309-2131.
